# Restricting carbohydrates to fight head and neck cancer—is this realistic?

**DOI:** 10.7497/j.issn.2095-3941.2014.03.001

**Published:** 2014-09

**Authors:** Rainer J. Klement

**Affiliations:** Department of Radiotherapy and Radiation Oncology, Leopoldina Hospital, Schweinfurt 97421, Germany

**Keywords:** Ketogenic diet (KD), head and neck neoplasms, diet, carbohydrate restricted (CHO restricted), nutritional support

## Abstract

Head and neck cancers (HNCs) are aggressive tumors that typically demonstrate a high glycolytic rate, which results in resistance to cytotoxic therapy and poor prognosis. Due to their location these tumors specifically impair food intake and quality of life, so that prevention of weight loss through nutrition support becomes an important treatment goal. Dietary restriction of carbohydrates (CHOs) and their replacement with fat, mostly in form of a ketogenic diet (KD), have been suggested to accommodate for both the altered tumor cell metabolism and cancer-associated weight loss. In this review, I present three specific rationales for CHO restriction and nutritional ketosis as supportive treatment options for the HNC patient. These are (1) targeting the origin and specific aspects of tumor glycolysis; (2) protecting normal tissue from but sensitizing tumor tissue to radiation- and chemotherapy induced cell kill; (3) supporting body and muscle mass maintenance. While most of these benefits of CHO restriction apply to cancer in general, specific aspects of implementation are discussed in relation to HNC patients. While CHO restriction seems feasible in HNC patients the available evidence indicates that its role may extend beyond fighting malnutrition to fighting HNC itself.

## Introduction

Head and neck cancer (HNC) is a collective term for cancers originating from the lip, oral and nasal cavity, paranasal sinuses, pharynx, larynx and trachea. Approximately 90% of HNCs are head and neck squamous cell carcinoma (HNSCC) originating from the mucosal lining (epithelium) of these regions.

Frequent comorbidities of HNCs include various feeding difficulties and malnutrition that are often aggravated by tobacco and alcohol abuse and a general unhealthy lifestyle^1^. At time of diagnosis up to 60% of all HNC patients present with improper nutritional status^2^^,^^3^, so that nutritional support becomes an important aspect in the treatment of these patients. A general recommendation is that even HNC patients who appear healthy should be counseled and advised to eat a high-calorie and high-protein diet^4^. In practice, however, the variety of available supplementary nutrition formulas and general inconsistent dietary advices for cancer patients^5^ pose a difficulty for deciding on the optimal diet for preventing muscle loss, improving the quality of life, reducing inflammation and withstanding therapy-induced side-effects. Many physicians seem unaware of the fact that besides the amount of caloric intake, the composition of the diet may have profound influences on these dietary goals. This is exemplified by a recent investigation of enteral and parenteral feeding practices in a Chinese university teaching hospital^6^ where only 2.1% of cancer patients received Supportan, a disease-specific high-fat nutrition formula that has been shown to improve nutritional and functional parameters in HNC patients compared to a standard formula^7^.

Like most aggressive tumors, HNCs exhibit a high rate of and dependence on glycolysis to meet their metabolic demands^8^^,^^9^. It has therefore been reasoned that diets restricted in carbohydrates (CHOs) could target the altered metabolism of such glycolytic tumors^10^^,^^11^. Indeed, there is some evidence that a ketogenic diet (KD), a high-fat low-CHO diet that leads to the elevation of circulating ketone bodies into the mM range, may not only impair tumor cell metabolism and growth, but also fight cachexia and therapy-induced side effects^12^^-^^14^.

In this review I am going to present three main rationales for the implementation of CHO restricted and KDs in HNC patients. Briefly, these are (1) targeting the altered tumor cell metabolism; (2) increasing the radio- and chemosensitivity of malignant cells while protecting normal cells; (3) accounting for the altered metabolism of the tumor-bearing host. Due to the various problems regarding food intake, the question remains whether CHO restriction is feasible in HNC patients. In the final part of this paper, I therefore address specific aspects and practical issues of such a dietary intervention.

## The sweet tooth of HNCs

A hallmark of HNC, like most cancers in general, is their high avidity for glucose uptake. Otto Warburg and his co-workers at the former Kaiser Wilhelm-Institute for Biology in Berlin were the first to quantify glucose uptake and energy generation in a large variety of animal and human tumors^15^^-^^19^. Using both *in vivo* and *in vitro* measurements, Warburg showed that compared to normal tissues, tumors would take up several times more glucose from the surroundings and ferment the majority of it to lactate even in the presence of sufficient oxygen that would normally suppress lactate production. This metabolic phenotype of increased glucose uptake and lactate production is therefore known as the Warburg effect or aerobic glycolysis since it also happens in normoxic conditions.

**Figure 1:**
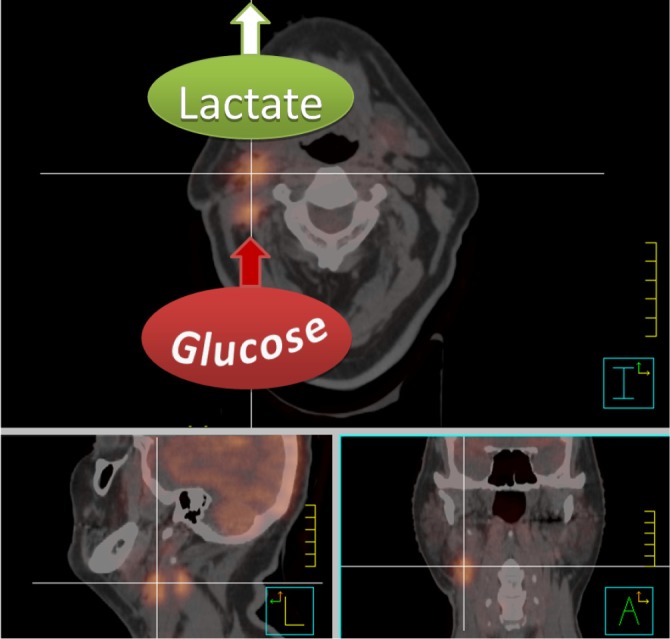
Fusion image of a radiotherapy planning CT and FDG-PET scan of a patient with a primary right-sided cT1 cN2b tonsillar squamous cell carcinoma after tonsillectomy. The high FDG uptake of the right lymph node conglomerates is indicative of highly glycolytic metastasis. Note, however, that FDG-PET only measures glucose uptake and conversion into glucose-6-phosphate, and can therefore not discriminate between lactate production or feeding of glycolysis intermediates and end products into the pentose phosphate pathway or citric acid cycle. The high lactate release which can be measured with other techniques such as magnetic resonance spectroscopy is, however, indicated for illustrative purposes since it is characteristic for aggressive metastasis.

The Warburg effect is the basic principle behind molecular imaging using positron emission tomography (PET) with the glucose analog 2-(^18^F)fluoro-2-deoxy-D-glucose (FDG). FDG is structurally similar to glucose except for the substitution of an OH group by the positron emitter ^18^F. Similar to glucose it therefore enters the cells through glucose transporters and gets phosphorylated by the enzyme hexokinase. Unlike glucose, however, FDG cannot be further metabolized after phosphorylation to FDG-6-phosphate and stays trapped inside the cell until it decays. The amount of assimilated FDG is quantified by the standardized uptake value (SUV) which expresses the ratio between the measured tissue activity and the injected activity standardized to body weight. In HNC FDG-PET in combination with computer tomography (PET/CT) has shown great benefit for tumor and lymph node staging, detection of an unknown primary tumor, radiation treatment planning, evaluation of therapy response and long-term surveillance^20^^,^^21^ (**Figure 1**). Furthermore, several studies have found that pretreatment tumor SUV—either as maximal SUV^22^^-^^25^ or combined with tumor volume into a total lesion glycolysis parameter^26^—is an independent significant predictor of local control, disease free and overall survival rates, while high lymph node SUVs were predictive for distant recurrence at 1 year^27^. Plasma glucose levels are able to falsify SUVs in highly glycolytic tumors^28^ which might account for negative results reported in some studies^29^.

The correlation between glycolytic rate and tumor aggressiveness is not only reflected on the side of glucose influx and hexokinase activity, but also at the final step of glucose fermentation in which pyruvate gets reduced to lactate. A large proportion of HNSCC overexpress lactate dehydrogenase 5, the enzyme that catalyzes this conversion, and this was correlated with poor prognosis^30^. Mechanistically, lactate is a key metabolite linking the Warburg effect to the other hallmarks of cancer such as sustained proliferative signaling, resisting cell death and activating invasion and metastasis^31^. Lactate production as measured by magnetic resonance spectroscopy may be a more sensitive parameter for the Warburg effect and thus tumor aggressiveness than FDG-PET^32^. There are several reasons why tumor lactate correlates with tumor aggressiveness. First of all, lactate acts as an antioxidant, protecting tumor cells not only from intrinsic reactive oxygen species (ROS) production, but also cytotoxic therapies. Indeed, high pretreatment lactate concentrations in head and neck tumors have been shown to increase the probability of metastatic spread in patients undergoing either postoperative or primary radiotherapy^33^^,^^34^. This is consistent with 1-(^11^C)-acetate PET measurements of tumor perfusion and oxidative metabolism in a small cohort of HNSCC patients that indicated that tumors utilizing predominantly glycolysis are more resistant to ionizing radiation than those with high cellular respiration^35^. Second, lactate (as well as pyruvate) seems able to stabilize hypoxia-inducible factor-1α (HIF-1α), a transcription factor that is also stabilized by hypoxia or oncogenic signaling and increases the expression of most glycolytic genes^36^. This provides a feed-forward loop in which tumor glycolysis sustains itself. Third, there is evidence showing that lactate promotes angiogenesis and metastatic spread^37^. Forth, lactate impairs the anti-tumor immune response by increasing the frequency of myeloid-derived suppressor cells and decreasing the cytolytic activity of NK cells^38^. Finally, lactate can be used as a fuel by some aerobic cancer cells^39^^,^^40^, and this may also apply to HNSCC^8^^,^^41^. Investigating cancerous oral mucosa slices from 40 patients, Curry *et al*.^41^ described a tumor compartment consisting of mitochondrial-rich proliferating cells staining positive for monocarboxylate transporter 1 (MCT1), the MCT isoform that normally mediates lactate uptake into respiring cells. This compartment was adjacent to non-proliferating, mitochondrial-poor tumor compartments staining positive for MCT4, the MCT isoform that passively transports lactate and protons out of glycolytic cells. Based on these findings the authors proposed a model of “metabolic compartmentalization” in which non-proliferating, glycolytic cells within the tumor stroma and epithelium would shuffle lactate and ketone bodies to mitochondrial-rich and highly proliferative epithelial stem-cell like cancer cells, thus fueling tumor growth and metastasis. However, besides the fact that no mechanism how stromal cells in the head and neck region would produce ketone bodies has ever been described, this study has not ruled out the possibility that MCT1-positive cells export lactate rather than importing it. Indeed, it was shown that in cells deficient in the p53 protein, MCT1 is up-regulated in response to hypoxia and able to work in the reverse mode, releasing lactate out of the cell, if glucose is abundant^42^. Therefore, as also argued recently by Doherty and Cleveland^43^, this model is probably no generalizable explanation for the observed relation between lactate and cancer aggressiveness.

## Genetic and epigenetic alterations promote aerobic glycolysis

On a molecular level HNC is a heterogeneous disease, complicating prognostication and the search for causative factors^44^^,^^45^. Yet the glycolytic phenotype appears to be a universal feature with good prognostic potential. Intriguingly, as **Table 1** shows, many of the most frequently mutated genes in HNSCC play a role in activating the metabolic switch towards aerobic glycolysis^46^^,^^48^^,^^49^^,^^51^^-^^53^. This metabolic shift also influences the epigenome by altering the availability of certain metabolic co-factors that are needed for epigenetic enzymes such as acetylCoA for histone acetylation or NAD+ for the class III histone deacetylase (HDAC) family of the sirtuins. In turn, epigenetic regulation of the expression of genes involved in metabolism may contribute to the altered tumor cell metabolism^54^. Epigenetic alterations are frequently observed in HNSCC and involve the methylation status of DNA (genome-wide hypomethylation and promoter region hypermethylation), post-translational histone modifications and post-transcriptional modifications through microRNAs^55^. Both DNA methylation and histone modifications such as methylation, acetylation or phosphorylation regulate gene expression by altering the chromatin structure between an open and closed form, thus allowing or blocking, respectively, the access of transcription factors to gene promoter regions. The activity of the enzymes catalyzing histone modifications is thereby not limited to the epigenetic regulation of gene expression, but also able to modify other non-histone proteins involved in cellular metabolism^54^.

**Table 1:** Common genetic mutations in HNSCC and their implication for tumor cell metabolism

Gene	Frequency (%)^45^	Implications for metabolism
Loss of function mutations/deletions		
*TP53*	50-80	Loss of p53 leads to nuclear and mitochondrial DNA instability, increased oxidative stress, decrease of OXPHOS and up-regulation of glycolysis (reviewed in^46^^,^^47^)
*NOTCH1*	14-15	Hypoactive Notch diminishes p53 levels and attenuates mitochondrial function, causing a switch to glycolysis and dependence on glucose^48^
*PTEN*	7	PTEN counteracts glycolysis by reversing the PI3K-mediated conversion of phosphatidylinositol1,4-biphosphate (PIP_2_) to phosphatidylinositol1,4,5-triphosphate (PIP_3_) that is required to activate Akt−mTOR signaling. Loss of PTEN therefore increases Akt activation. PTEN also counteracts glutaminolysis by reducing glutaminase levels through a PI3K-independent pathway^49^
Gain of function mutations/amplifications		
*PIK3CA*	6-20	PIK3CA encodes p110α, an isoform of the 110-kDa catalytic subunit of the class 1A phosphatidylinositol-3-kinase (PI3K). The PI3K−Akt−mTOR pathway is one of the most frequently hyperactivated signaling cascades in tumor cells. Enhanced Akt signaling induces a Warburg phenotype and increases the coupling of glycolysis to the mitochondrial citric acid cycle which yields intermediates for biosynthetic pathways and NADH as the primary electron donor for OXPHOS (reviewed in^50^)
*HRAS*	4-5	HRAS encodes the small GTPase H-Ras, a member of the Ras superfamily of enzymes that become active when bound to GTP. Besides other pathways important for cell survival and proliferation, Ras-GTP directly acivates PI3K p110. Oncogenic H-Ras activation diminishes mitochondrial respiration, rendering transformed cells depend on glucose to fuel glycolysis^51^

The tumor suppressor p53 is one example since its activity and stability can be regulated through lysine methylation and acetylation by various histone-modifying proteins. Although epigenetic silencing of p53 seems rare in HNSCC^56^, the *TP53* gene is found to be mutated in approximately 60% of all HNSCC, and its protein product p53 inactive in another 20% due to degradation by the human papilloma virus oncoprotein E6^44^. A loss of p53 promotes aerobic glycolysis, increases the flux through the pentose phosphate pathway (PPP) and down-regulates mitochondrial oxidative phosphorylation (OXPHOS) through various pathways (reviewed in^46^^,^^47^). Furthermore, p53 deficiency increases the levels of ROS which promotes further DNA mutations and an up-regulation of glycolysis via hypoxia-independent stabilization of HIF-1α^57^. In this way HIF-1α protects tumor cells against steady-state oxidative stress through the production of lactate and pyruvate by glycolysis and regeneration of reduced glutathione (GSH) via NADPH production in the oxidative PPP^37^^,^^58^. This protection probably extends to the oxidative stress induced by chemotherapy and ionizing radiation as shown in HNSCC xenografts^59^^,^^60^. It breaks down upon glucose deprivation, leading to ROS-induced cell death^61^^-^^64^. Interestingly, a p53-independent overexpression of the p53 target gene *TP53*-induced glycolysis and apoptosis regulator (TIGAR) is often observed in tumor cells, causing an up-regulation of the oxidative and non-oxidative PPP which are important for the production of NADPH and ribose-5-phosphate (an anabolic intermediate needed for nucleotide production), respectively^47^. Another gene related to the PPP is transketolase-like-1 (TKTL1). TKTL1 seems frequently overexpressed in HNSCC due to promoter hypomethylation and increases aerobic glycolysis and HIF-1α accumulation^65^. Consistent with this, a high degree of staining for TKTL1 has been linked to a significantly shorter disease-specific survival in laryngeal^66^ and oral^67^ SCC patients.

Finally, p53 plays an important role in mitochondrial DNA (mtDNA) repair and stability, and some studies have shown associations between mutant p53 and mtDNA mutations^68^^,^^69^. MtDNA mutations may cause mitochondrial dysfunction since the mtDNA encodes 20% of the OXPHOS genes^70^. This may also impair nuclear DNA stability by leading to an increased release of ROS into the cytosol^70^ and a retrograde response involving downregulation of the nuclear DNA repair protein APE1^71^. About 24%-47% of HNSCC are estimated to harbor pathogenic mtDNA mutations that may alter mitochondrial function through aberrant transcription, translation or replication^68^^,^^69^. High steady state levels of ROS caused by mitochondrial dysfunction would have to be neutralized by a high rate of glycolysis else they induce tumor cell death^61^^-^^64^^,^^72^. Zhou and colleagues^68^^,^^73^ have provided evidence that mtDNA mutations in HNSCC contribute to the Warburg effect via ROS-induced stabilization of HIF-1α, although Challen *et al*.^74^ found no association between mtDNA mutations and the expression of four HIF-1α target genes. The complex interaction between the nucleus and the hundreds to thousands of mitochondria in the cell may explain part of the controversy about the role of mtDNA mutations as drivers or bystanders of tumor progression^74^. However, there is evidence that mtDNA mutations and a dysfunctional TCA cycle induce a compensatory up-regulation of glycolysis^10^^,^^11^. This relates to the original hypothesis by Otto Warburg that cancer and aerobic glycolysis are caused by respiratory insufficiency^75^. Some rare forms of familial and sporadic head and neck paragangliomas indeed arise due to a dysfunctional TCA cycle. These tumors are caused by germline loss-of-function mutations in the *SDHC* gene encoding the C subunit of the TCA cycle enzyme succinate dehydrogenase. As a consequence succinate accumulates in the mitochondria and leaks out into the cytosol where it inhibits certain prolyl hydroxylase enzymes leading to stabilization of HIF-1α and aerobic glycolysis^76^. Furthermore Yang and colleagues provided evidence that oncogenic *HRAS* transformation impairs mitochondrial respiration which subsequently increases glycolysis without compromising mitochondrial mass or content^51^.

## Vulnerability of HNC cells to CHO restriction

The shift in HNC metabolism towards aerobic glycolysis concurrently provides high energy production, cytotoxic stress resistance, building blocks for rapid proliferation and the ability to migrate out of the cell compartment. However, this comes at the expense of metabolic flexibility. The genetic and epigenetic alterations found in HNC cells in conjunction with mitochondrial defects and hypoxia leave these cells extremely dependent on a steady supply of nutrients, especially glucose. Numerous *in vitro* experiments have shown that cancer cells are particularly vulnerable to glucose (and glutamine) restriction^63^^,^^64^^,^^77^^-^^81^. Although glutamine also plays a role as an energetic and anaplerotic substrate in cancer cells it seems to be less important than glucose in HNSCC^9^. The biophysical theory of quantum metabolism predicts that cancer cells relying on glycolysis outperform normal metabolically flexible cells only as long as glucose is abundant and dominates other metabolic fuels^82^^,^^83^. This implies to impose limitations on glucose abundance and increase the diversity of alternative substrates as a therapeutic strategy^83^. Dietary CHO restriction is a non-toxic approach of reducing the supply of blood glucose to cancer cells and increasing the utilization of fatty acids and ketone bodies in normal cells^10^^,^^12^^,^^84^^,^^85^. In particular, KDs may have beneficial effects when used as a supportive dietary manipulation in cancer patients. A KD mimics the metabolism of fasting without restricting energy intake, mainly by replacing CHOs with fat. The restriction of CHOs seems to be responsible for most of the beneficial effects of calorie restriction. A moderate to severe restriction of CHOs without limiting energy intake is therefore a good alternative to a calorie restricted diet when weight loss must be prevented^14^^,^^86^. One main rationale for dietary CHO restriction in cancer patients is its ability to simultaneously exploit several of the following factors underlying tumor glycolysis (**Table 2**):

**Table 2:** Targeting tumor glycolysis through carbohydrate restriction

Factors promoting glycolysis	CHO restriction as a metabolic strategy to target these factors
High blood glucose levels (Up-regulation of glycolytic enzymes)	Blood glucose ↓, fatty acids ↑ (Fatty acids inhibit key glycolytic enzymes)
Hypoxia	Blood glucose ↓, ketones ↑ (Poor nutrient supply to hypoxic cells; hypoxic cells unable to efficiently oxidize ketones)
Oncogenic signaling (Insulin/IGF-1−PI3K−Akt−mTOR)	Blood glucose ↓, insulin ↓ (Counteracts PI3K pathway; also activates AMPK, inhibiting mTOR)
	Ketones ↑ (Class I and II HDAC inhibitors)
Inflammation (High blood glucose levels and ROS promote inflammatory cytokine release)	Blood glucose ↓ (Decreases ROS and inflammation)

### CHO restriction down-regulates glycolysis

CHO restriction and its replacement with fat elevate free fatty acids and decreases glucose concentrations in serum. Fatty acids—in particular the saturated ones—have been shown to inhibit the key glycolytic enzymes hexokinase, phosphofructokinase, pyruvate kinase and lactate dehydrogenase^87^. While even low physiological blood glucose levels should not be rate-limiting to glucose influx into tumor cells since their glucose transporters have low Km values and therefore a high affinity for glucose, this situation may be different in poorly vascularized tumor areas as glucose concentrations decrease along their diffusion paths. FDG-PET studies confirm that a KD is able to inhibit tumor glycolysis in some cancer patients^88^^-^^90^. Two recent studies in mice showed that a KD lowered lactate production and resulted in less tumor growth^38^^,^^40^. Importantly, Schroeder *et al*.^91^ recently described a KD-induced reduction of lactate levels in a small group of HNSCC patients using implanted microdialysis catheters. For 4 days the patients received a diet consisting of solely meat, fish, eggs, salad, cheese and sausages; these foods were grinded for patients with dysphagia and tolerated very well (Ursula Schroeder, private communication). This short-term KD decreased blood glucose levels and induced a decline of intra-tumoral lactate levels that was far greater than in normal mucosa. Although the reduction of blood glucose concentrations may be facilitated and increased with concurrent calorie restriction^11^, this is no option for HNC patients with a high risk for weight loss.

### CHO restriction is especially problematic for hypoxic cells

The role of hypoxia in up-regulating glycolytic enzymes and glucose entry into the cells is well established. Already Warburg was aware of the poor capillary network of tumor tissue and hypothesized that tumors are more vulnerable to simultaneous glucose and oxygen deprivation due their worse “channels of supply”^19^. In principle, a lowering of blood glucose levels might cut some of the chronically hypoxic cells lying far from blood vessels completely off their supply. If CHOs are restricted severely enough, the liver also starts to produce larger amounts of ketone bodies that serve as a high quality fuel for normal tissues, in particular the brain and muscles^92^. Although measurements in HNSCC patients have shown that their tumors take up ketone bodies, the absolute amounts were small and their metabolic fate was not determined^8^. Even if HNSCC would have the necessary enzymes to utilize ketone bodies—which seems not the case for many other tumor cells^93^^-^^96^—utilization of ketones requires oxygen and is therefore impaired in large parts of the tumor^40^. Thus, a lowering of blood glucose levels will have a much harder impact on hypoxic tumor cells than on normal cells that are metabolically flexible and possess an intact nutrient supply network.

### CHO restriction inhibits oncogenic signaling

CHO restriction has the ability to counteract signaling through the phosphatidylinositol-3 kinase−Akt−mammalian target of rapamycin (PI3K−Akt−mTOR) pathway. This pathway is activated by insulin and growth factors such as insulin-like growth factor-1 (IGF-1) and its effect is, among others, an up-regulation of glycolysis^50^^,^^97^. The complexity of the IGF signaling network, tyrosine kinase receptor crosstalk as well as autocrine activation of non-targeted receptors all provide resistance mechanisms against overly specific tyrosine kinase inhibitors that additionally often induce systemic side effects^98^^,^^99^. In contrast, CHO restriction is a non-toxic strategy to simultaneously target the same molecular pathways that are individually targeted with pharmaceutical drugs.

Conflicting and often negative results concerning an association between IGF-1 and cancer have been reported for a variety of cancers including HNC^98^. This leaves insulin, hyperglycemia and inflammation as more plausible mediators of the well-established metabolic syndrome-cancer connection^100^. A recent study has extended this link to HNC by showing that obesity is an independent risk factor for disease-specific mortality from early stage oral SCC when the influence of cancer-associated weight loss is accounted for^101^. Thus, in early stage HNSCC insulin inhibition through CHO restriction may be beneficial against tumor glycolysis and progression. CHO restriction also increases AMP kinase (AMPK) activity, an intracellular energy sensor. Although AMPK activation can acutely up-regulate glycolysis in some cells, in the longer term it acts as an “anti-Warburg” tumor suppressor and inhibits mTOR signaling^102^. AMPK has therefore emerged as an attractive anti-cancer target that is tried to be activated using anti-diabetic drugs such as metformin^99^^,^^103^.

Insulin inhibition may be less effective in later stages of tumor progression as genetic and epigenetic alterations accumulate and chronically activate the PI3K−Akt−mTOR pathway^104^. Work from Adrienne Scheck and co-workers implies, however, that KDs exert effects extending beyond those of insulin inhibition by inducing global changes in tumor gene expression that counteract glycolysis and tumor growth^105^^,^^106^. Interestingly, β-hydroxybutyrate and, to a lesser extent, acetoacetate act as class I and II HDAC inhibitors^107^^,^^108^. As such, ketone bodies may alter signaling pathways in HNC through epigenetic and non-epigenetic mechanisms, providing novel anti-cancer functions. For instance, chemically induced chromatin acetylation through the HDAC inhibitor Trichostatin A has been shown to significantly impair the proliferation of HNSCC spheres and to reduce the fraction of cancer stem cells^109^. Furthermore, findings relating the overexpression of HDAC 2 to post-translational HIF-1α stabilization in oral SCC imply a role for HDAC inhibitors as “anti-Warburg” agents^110^. It must be noted, however, that ketone bodies are less potent than other clinically employed HDAC inhibitors suggested for the treatment of HNSCC, so that their anti-cancer effects relating to their role as HDAC inhibitors remain to be elucidated.

In summary CHO restriction and KDs in particular exert systemic effects on oncogenic signaling pathways that counteract tumor glycolysis but—owing to the complexity of the signaling networks involved and the large genetic heterogeneity of HNC tumors—need to be further investigated.

### CHO restriction targets inflammation

The relationship between inflammation and HNSCC becomes apparent from a Hungarian study showing an increased prevalence of oral inflammatory, premalignant and cancerous lesions among diabetics compared to healthy controls^111^. These authors also found that with 14.6% and 9.7%, respectively, the prevalence of diabetes and elevated blood glucose levels (>6.1 mmol/L) was significantly higher in 610 oral carcinoma patients than in a tumor-free control group. High blood glucose levels promote the release of inflammatory cytokines and ROS from monocytes and macrophages in a dose-dependent manner^112^^,^^113^; both inflammatory cytokines and ROS are activators of HIF-1α and therefore glycolysis. The connection between inflammation and high blood glucose levels is also seen in cancer cachexia syndrome^12^. In fact, already in 1885 Ernst Freund described signs of hyperglycemia in 70 out of 70 cancer patients, which led him to conclude that the abnormally high blood sugar content would be necessary for the existence of a carcinoma^114^. Along these lines, hyperglycemia is now an established predictor of poor survival in a variety of cancers^115^^-^^122^.

It therefore seems prudent to limit high blood glucose spikes that may particularly occur with nutritional support containing simple sugars. In a retrospective analysis of data from the RTOG 90-03 trial involving 1,073 HNSCC patients, Rabinovitch *et al*.^123^ clearly showed that baseline nutrition support before definitive radiotherapy significantly decreased locoregional control and 5-year overall survival by an absolute amount of 28% and 33%, respectively, despite diminishing treatment-related weight loss. After adjusting for a range of other prognostic factors through recursive partitioning analysis, baseline nutrition support remained as a highly significant (*P*<0.0001) risk factor for locoregional failure and death. Although detailed information on the composition of the nutritional support was lacking, it is clear that the standard nutrition support formulas used at that time contained a high percentage of high glycemic CHOs which would lead to blood sugar and insulin spikes and fuel inflammation. It is also clear from these results that the treating physician should not only focus on the quantity of calories, but that their quality might be more important for patient survival.

## CHO restriction during radiation treatment

**Figure 2:**
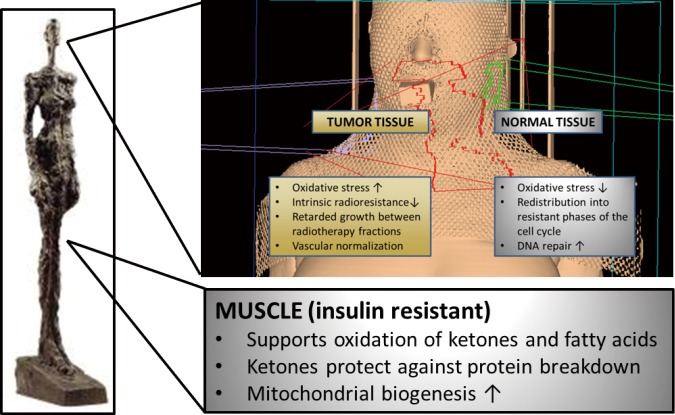
Putative effects of CHO restriction on normal and tumor tissue. During radiotherapy CHO restriction may induce a differential stress response between normal and tumor cells such that the former experience protection from and the latter sensitization to ionizing radiation. Additionally, through the elevation of ketone bodies and fatty acids, CHO restriction helps to conserve muscle tissue.

The optimal treatment of HNC requires a multidisciplinary approach in which radiation therapy constitutes the major modality besides surgery^124^. Unfortunately HNCs often exhibit increased radioresistance that is linked to their glycolytic phenotype^58^. These resistance mechanisms might be targeted by dietary modulation. Preclinical data indicate that calorie and CHO restriction during cancer therapy differently alter the stress resistance of tumor and normal tissue such that the former experiences sensitization to and the latter protection against ionizing radiation and chemotherapy^72^^,^^106^^,^^125^^-^^128^. Central to this differential stress resistance is the energy sensing network consisting of AMPK, the NAD+-dependent class III HDAC silent mating type information regulation 2 homologue 1 (SIRT1), peroxisome proliferator-activated receptor α (PPARα) and the transcription factor peroxisome proliferator-activated receptor γ coactivator α (PGC-1α)^14^^,^^129^. In normal human tissue, this network is generally activated through any stress that decreases blood glucose levels and activates lipid mobilization and oxidation: calorie restriction, fasting, exercise or—as emerging evidence indicates—CHO restriction. AMPK/SIRT1/PPARα/PGC-1α signalling not only serves to up-regulate mitochondrial biogenesis and respiration, but also functions to “clean up” cells via autophagy and to protect them against inflammation and DNA damage. Thus, proper activation of this network would offer protection to normal cells during radio- and chemotherapy (**Figure 2**).

With few exceptions, normal tissues readily oxidize ketone bodies thereby decreasing the mitochondrial NADP+/NADPH ratio. This in turn increases the amount of reduced GSH available for scavenging ROS^130^. This antioxidative property of ketone bodies would not benefit tumor cells which are unable to metabolize them due to a lack of the necessary enzymes^93^^-^^96^ or hypoxia^40^. In contrast, the HDAC inhibiting activity of ketones could be useful against HNSCC stem cells which typically exhibit the highest radioresistance^109^.

CHO restriction also up-regulates lipid oxidation which increases the intracellular NAD+/NADH ratio and thus amplifies the NAD+-dependent activity of SIRT1^129^. SIRT1 enhances the repair of single and double strand breaks that are induced by ionizing radiation, in this respect acting as a tumor suppressor^131^. SIRT1 also interacts with p53 and forkhead box O (FOXO) transcription factor proteins to induce cell cycle arrest in order to keep cells from transitioning into replicative phases of the cell cycle in which they are most vulnerable to cytotoxic insults. Thus, CHO restriction before a radiotherapy session could be employed to redistribute normal cells into a non-dividing resistant state. These SIRT1-mediated protection mechanisms are probably less pronounced or defective in HNC cells. For instance nuclear SIRT1 expression generally seems to be lower in HNSCC than in normal mucosa^132^, suggesting an impaired global genome nucleotide excision repair through repression of xeroderma pigmentosum C expression^133^. Furthermore phosphorylation of FOXO transcription factors through Akt leads to their exclusion from the nucleus and cytosolic degradation. Thus constitutive activation of Akt as well as loss of p53 in HNSCC tumors would disrupt FOXO-mediated transcription of DNA repair genes and induction of cell cycle arrest upon CHO restriction.

CHO restriction may also impair tumor re-growth during radiotherapy fractions and delay the accelerated proliferation that is known to start in HNSCC at some point during radiation treatment. We have previously reviewed the wealth of preclinical data showing that CHO restriction alone delays tumor growth in a variety of tumor models^12^. Most of these studies tested KDs, and *in vitro* data indicate that ketone bodies themselves can have anti-proliferative effects on some tumor cells^134^^,^^135^. Unfortunately, most human studies assessing growth inhibition through KDs have specifically focused on advanced stage astrocytoma patients that have a particularly bad prognosis^88^^,^^89^^,^^136^. In addition, subject numbers in the studies to date are small, reducing the statistical reliability of the results. Nevertheless some hints for a reduction of tumor cell proliferation with CHO restriction in extra-cranial tumors have been found in small pilot trials^90^^,^^137^.

Some preclinical studies have further shown that CHO restriction either in the form of overall calorie restriction^138^^-^^140^ or an unrestricted KD^141^ may target the vascular endothelial growth factor pathway that is also targeted by the drug bevacizumab (Avastin) for which clinical benefits have been shown when combined with radio- and/or chemo-therapy in HNSCC^142^. VEGF inhibition is employed in order to normalize the tumor vasculature and radiosensitize tumor tissue by facilitating the delivery of oxygen and chemotherapeutic drugs. Along these lines, hyperbaric oxygen therapy (HBOT) has been employed to enhance the efficacy of radiotherapy. A Cochrane review has concluded that HBOT during radiotherapy significantly lowers the risk of tumor recurrence at one and five years in HNSCC, but at the expense of increased normal tissue injury and central nervous system oxygen toxicity. In this context it is interesting to note that two preclinical studies provide evidence that ketone bodies might have synergistic effects with HBOT^143^ and significantly delay the onset of HBOT-induced seizures^144^.

In summary, there is evidence that CHO restriction acts as a cytotoxic sensitizer in tumor tissue while simultaneously protecting normal tissue which supports its implementation during standard treatment for HNC. The main effects are summarized in **Figure 2**, and a more thorough review of the underlying mechanisms has recently been published^14^.

## CHO restriction to positively influence body composition

Unintentional weight loss is a common problem in HNC patients. It is often present already at the time of diagnosis and indicates malnutrition or an early cachectic state. Weight loss is usually influenced by disease- and treatment-associated problems with food intake such as dysphagia, xerostomia, mucositis and anorexia. Cachexia differs from malnutrition or physiological states of low calorie intake in that it is characterized by a progressively increasing change in whole-body metabolism that induces a continuous loss of skeletal, but not visceral, tissue. It is driven by a complex interaction between the tumor and its host involving a multitude of heterogeneous factors that are, however, mostly connected to chronic systemic inflammation^145^. Muscle wasting is mainly responsible for the negative effects of cancer-related weight loss. These include declines in strength, quality of life and tolerability of cancer treatment. A simple and easy-to-obtain indicator of muscle mass and strength exists in the form of the phase angle (PA) measured by tetrapolar bioelectrical impedance analysis^146^. The PA is determined by tissue cellularity, hydration and membrane potential and therefore is useful to assess malnutrition at the cellular level. Consistently, low PAs have been shown to strongly predict outcome in cancer patients^147^^-^^149^. Bioelectrical impedance measurements have shown that already at early stages of disease, i.e., with normal BMI and minimal previous weight loss, HNC patients exhibit a significantly lower PA than age-matched healthy controls that is not explainable by an altered hydration status^150^. This sign of “sub-clinical malnutrition” therefore seems to occur early in the progression of disease and may be connected to the early systemic insulin resistance that has been described in a variety of cancer patients^151^^-^^154^. Pro-inflammatory cytokines are thought to play a causal role in this insulin resistance, similar to adipokines in obesity. Insulin resistance has important consequences for whole-body metabolism: In the liver, the rate of gluconeogenesis increases, utilizing lactate from the tumor, alanine from muscle and glycerin from lipolysis as substrates. As an energy-consuming process gluconeogenesis might therefore contribute to weight-loss. In liver and skeletal muscle, glucose uptake and storage are inhibited^155^^-^^157^, and some^156^^,^^158^, but not all^159^, studies have found a decreased glucose oxidation rate. In contrast, gradually increased lipid oxidation rates have been measured in weight-stable and weight-losing patients^158^^,^^160^, and even exogenous glucose ingestion was not able to reverse lipid oxidation to normal levels^159^. Finally, with disease progression the prolonged inflammatory state further takes its toll on muscle as amino acids are increasingly broken down and used for the synthesis of acute-phase proteins in the liver.

These metabolic alterations indicate a general dysfunction in the utilization of glucose and an increased demand on fat as an energy source. This is quite contrary to the metabolism of tumor tissue that mainly utilizes glucose. It has therefore been recommended to account for these metabolic differences between the tumor and its host through either a high-fat low-CHO diet or a KD^12^^,^^13^ (**Figure 2**). Unfortunately, clinical studies on these diets are rare and concentrate on advanced-stage patients. In a randomized controlled trial on patients with advanced gastrointestinal cancer undergoing chemotherapy, Breitkreuz *et al*.^161^ supplemented the conventional diet of 12 patients with a drink containing 66% energy from fat, while 11 patients remained on their conventional diet. Although there were no significant differences between the groups with respect to non-nitrogenous energy intake, the treatment group had gained weight at 4 and 8 weeks and retained their body cell mass, while the control group continued to lose weight. Fearon *et al*.^162^ administered a KD containing 70% energy from medium chain triglycerides and supplemented with β-hydroxybutyrate to five extremely cachectic patients (mean body weight 38.6 kg). After 1 week, the patients had regained approximately 2 kg body weight and improved their physical performance status. However, in this study there was no change in nitrogen balance that would have explained the significant weight gain. Other findings suggest that the physiological role of ketone bodies in the conservation of muscle tissue during prolonged starvation is retained even in cachectic patients^163^. Rat studies provide evidence that physiological levels of ketone bodies diminish muscle catabolism by inhibiting the oxidation of branched chain amino acids^164^ and reducing the release of the gluconeogenic amino acid alanine^165^. We^166^ and others^167^^,^^168^ have shown that several weeks of a KD combined with ample protein intake increased the muscle mass in recreational and top-level athletes, respectively, despite a small overall weight loss.

In HNC patients, even “sufficient” energy and protein intakes have been shown to be insufficient for preventing significant weight and lean tissue loss during treatment^169^. Accordingly a recent position paper from a European School of Oncology Task Force states that “every effort should be made to prevent muscle loss rather than relying on attempts to regain what has been lost”^170^. Following this statement the usage of ketone esters or KDs could be tried as part of such an effort.

## Discussion: is CHO restriction in HNC patients realistic?

Despite the evidence outlined above showing how CHO restriction counteracts tumor glycolysis, accounts for the altered metabolism of the tumor-bearing patient and may even improve the tolerability of radiation treatment, some authors still question the scientific rationale for the KD and deny any possible benefits^171^. This probably reflects a general skepticism towards the implementation of low CHO or KDs that are “extreme” in the sense that they go against official standard recommendations of the food agencies. In this context it should be noted, however, that the 2006 guidelines of the European Society for Enteral and Parenteral Nutrition (ESPEN) stated that the observations on altered patient metabolism “may be taken to support recommendations to increase the fat/CHO ratio in feeding cancer patients”^172^. Arguments against a KD include the fear of “ketoacidosis”, excessive weight loss, high cholesterol levels, kidney problems or the belief that the brain depends on a certain CHO intake^171^. However, none of these fears and assumptions are justified in light of the evidence from the literature^13^^,^^173^^-^^175^. Quite to the contrary, the literature on cancer patients suggests that even in very advanced stages low CHO diets combined with moderate to high protein intake may be anti-catabolic^162^, without serious side effects^90^^,^^136^^,^^176^ and able to improve blood parameters and some aspects of quality of life^176^. It is clear, however, that once a cachectic state has been reached any nutritional support will be insufficient in stopping the weight loss^170^^,^^177^. Furthermore the ability to develop ketosis might be impaired in a large fraction of cachectic patients^163^. A low-CHO diet as a supportive treatment for HNC should therefore be offered as soon as the patient has been assessed for malnutrition, and then be implemented before weight loss becomes irreversible^170^.

**Figure 3:**
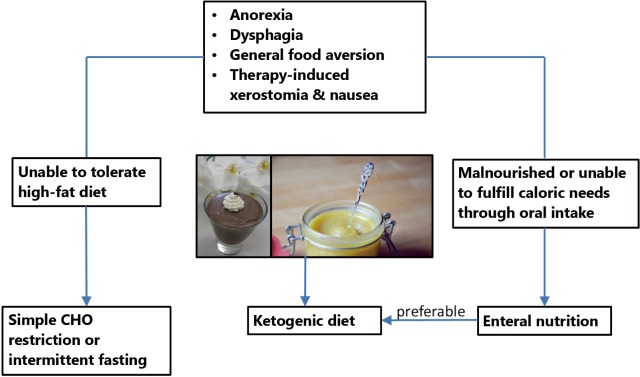
Flow chart showing the proposed implementation of a low CHO diet for the HNC patient. The pictures show foods compatible with a ketogenic diet that have a creamy texture and thus are easy to swallow.

Studies so far have shown that individual dietary counselling during radiotherapy of HNC is more effective than standard or no dietary advice for preventing long-term weight loss and improving quality of life, although the effects of these interventions on mortality have not been assessed^178^. This would be important, however, since nutrition support using standard high-CHO formulas has been shown to increase mortality rates despite better preservation of body weight during therapy^123^. Already in 1979 Donaldson and Lennon warned against this danger of nutrition support in the HNC patient^2^. From the mechanistic insights outlined here, this is now understandable. **Figure 3** therefore presents a possible flow chart for individual counselling of HNC patients when the goal is to implement a low-CHO diet in order to minimize the risk of inducing high blood glucose levels and spurring tumor growth. First of all any individual problems with food intake and the nutritional status have to be assessed. Common problems such as dysphagia, xerostomia or odynophagia can often be addressed by blending foods and using liquid supplements^1^. The high-fat nature of low CHO diets allows one to offer a variety of foods with a creamy texture that facilitate swallowing. If a patient is not yet malnourished, but unable to tolerate or accept a diet high in fat, then simple CHO restriction, the avoidance of high glycemic CHOs or intermittend fasting are options to try^14^. If, however, the patient has been classified as malnourished or is unable to meet his or her caloric needs through oral intake, supplemental tube feeding should be considered according to recommendations from the literature^4^. This can be seen as an opportunity for implementing low-CHO high-fat feeding since suitable feeding formulas are available for prescription. Thereby it seems preferable to try a KD based on the beneficial properties of ketone bodies outlined in this paper. In addition or as an alternative, ketone esters may be used to induce “therapeutic ketosis” with ketone levels in the 2-7 mM range lasting for several hours upon ingestion^179^. To further optimize ketosis, it may be considered that the ability of different protein sources to elevate blood glucose levels via gluconeogenesis negatively correlates with the fraction of their amino acids utilized in anabolic pathways, also known as net nitrogen utilization^180^. Accordingly, protein sources with high net nitrogen utilization such as eggs and meat or the Master Amino Acid Pattern supplement^180^, a blend of the essential amino acids with a net nitrogen utilization of 99%, might be preferred, although the effects of such strategies on ketosis have not yet been systematically evaluated. Finally, to fully exploit the potential of CHO restriction in HNC it should be used within a multimodal approach combined with anti-inflammatory^170^ and anti-cancer metabolic^11^ therapy. This may further include nutraceuticals such as ω-3 fatty acids as well as resistance training that has shown great potential in the recovery of muscle mass and strength after HNC therapy^181^.

In conclusion, CHO restriction in the HNC patient seems feasible and therefore realistic, but requires additional time and effort as it has to be tailored towards the individual patient. This, however, is a general problem in HNC patients, and efforts on nutritional counselling generally seem to pay off. Clearly, the tolerability of and response to CHO restricted diets is also individual and some patients reach ketosis more easily than others. Hopefully future studies will show which patients benefit most from CHO restriction. Currently a phase I clinical trial (NCT01975766) at the university of Iowa recruits HNSCC patients to investigate the safety of a KD plus concurrent chemoradiation with a secondary outcome being progression-free survival. Until the results are published, this paper hopefully encourages physicians to make their own experience with offering CHO restriction to their patients as a non-toxic approach to fight HNC.
